# Bioorthogonal Chemistry Approach for the Theranostics of GRPR-Expressing Cancers

**DOI:** 10.3390/pharmaceutics14122569

**Published:** 2022-11-23

**Authors:** Alice D’Onofrio, Francisco Silva, Lurdes Gano, Paula Raposinho, Célia Fernandes, Arkadiusz Sikora, Monika Wyczółkowska, Renata Mikołajczak, Piotr Garnuszek, António Paulo

**Affiliations:** 1 Centro de Ciências e Tecnologias Nucleares, Instituto Superior Técnico, Universidade de Lisboa, Campus Tecnológico e Nuclear, Estrada Nacional 10, Km 139.7, 2695-066 Bobadela LRS, Portugal; 2 Departamento de Engenharia e Ciências Nucleares, Instituto Superior Técnico, Universidade de Lisboa, Campus Tecnológico e Nuclear, Estrada Nacional 10, Km 139.7, 2695-066 Bobadela LRS, Portugal; 3 National Centre for Nuclear Research, Radioisotope Centre POLATOM, 05-400 Otwock, Poland

**Keywords:** click chemistry, GRPR antagonist, theranostics, tetrazine, PRRT

## Abstract

Several gastrin-releasing peptide receptor (GRPR) antagonists with improved in vivo behavior have been recently developed and tested in the clinic. However, despite the generally mild side effects of peptide receptor radionuclide therapy (PRRT), toxicity has been observed due to high doses delivered to nontarget tissues, especially in the kidneys and pancreas. Previous experiences with radiolabeled peptides opened a unique opportunity to explore GRPR pretargeting using clickable bombesin antagonists. Toward this goal, we used clickable DOTA-like radiocomplexes which have been previously evaluated by our group. We functionalized a potent GRPR antagonist with a clickable TCO moiety using two different linkers. These precursors were then studied to select the compound with the highest GRPR binding affinity and the best pharmacokinetics to finally explore the advantages of the devised pretargeting approach. Our results provided an important proof of concept toward the development of bioorthogonal approaches to GRPR-expressing cancers, which are worth investigating further to improve the in vivo results. Moreover, the use of clickable GRPR antagonists and DOTA/DOTAGA derivatives allows for fine-tuning of their pharmacokinetics and metabolic stability, leading to a versatile synthesis of new libraries of (radio)conjugates useful for the development of theranostic tools toward GRPR-expressing tumors.

## 1. Introduction

G protein-coupled receptors (GPCR) are a heterogeneous family of transmembrane receptors clinically relevant as imaging or therapeutic targets in a huge variety of diseases such as respiratory conditions, mental health disorders and cancer [1]. The bombesin receptor family is a class of GPCR that was discovered after the isolation of the 14-peptide bombesin (BBN) from the European fire-bellied toad Bombina Bombina, which strongly interacts with three mammalian receptors: the neuromedin B receptor (NMBR or BB1), the gastrin-releasing peptide receptor (GRPR or BB2) and the bombesin receptor subtype-3 (BRS-3 or BB3). The endogenous ligands of these receptors in humans are the decapeptides Neuromedin B and C and the 27-peptide GRP. Despite possessing different lengths, a strongly conserved sequence, composed of the last seven to nine C-terminal amino acids, seems to be crucial for biological activity [2].

The subtype GRPR is particularly relevant for the development of anticancer therapies and its overexpression has been reported in several cancer types such as small lung cell, head/neck squamous cell, colon, glioblastoma, breast and prostate cancer [3]. GRPR is physiologically highly expressed in the pancreas, and at lower levels is also expressed in the colon, breast, prostate, as well as in the central nervous system [4]. Due to the recent FDA approval of peptide-based radiopharmaceuticals targeting the somatostatin receptor and the prostate-specific membrane antigen (PSMA), the development of GRPR-targeting agents suitable for clinical translation has been thriving [5].

The main limitation to the clinical use of radiolabeled peptides is the metabolic stability and, in recent years, several groups have been intensely working toward BBN analogs with improved in vivo behavior [6,7]. However, despite their high binding affinities, the use of GRPR agonists has been associated with chronic desensitization, downregulation of receptor expression and consequent biosafety issues when administered at higher peptide doses [8]. Nonetheless, GRPR antagonists have demonstrated superior pharmacokinetic properties and high tumor accumulation in several in vivo models, emerging as a valuable alternative in the development of GRPR-targeting radiopharmaceuticals [9,10,11,12,13]. In addition, GRPR antagonists demonstrated in vitro and in vivo antiproliferative effects in several cancer models and the inhibition of tumor growth through different mechanisms, namely by interfering with the epidermal growth factor (EGF) and the vascular endothelial growth factor (VEGF) signaling pathways [14,15,16,17].

Several GRPR antagonists resulting from modifications to the C-terminal sequence of bombesin have been developed and have been recently extensively reviewed [18]. Some of the compounds developed have been already tested in the clinic, including those bearing the gamma amino acid statine (Sta) instead of leucine (Leu) at position 13, a modification that seems to improve both the binding affinity and the stability, achieving excellent in vivo properties when compared to the potent agonist [^111^In]In-AMBA [10,19]. Furthermore, in a recent study, the additional replacement of L-Trp at position 8 with an α-methyl-L-tryptophan (α-Me-L-Trp) also led to the development of two novel derivatives bearing a 2,2′,2′′,2′′′-(1,4,7,10-tetraazacyclododecane-1,4,7,10-tetrayl)tetraacetic acid (DOTA) and a 1,4,7,10-tetraazacyclododececane,1-(glutaric acid)-4,7,10-triacetic acid (DOTAGA) moiety, with even better in vivo stability and overall preclinical performance [12].

Although the side effects of peptide receptor radionuclide therapy (PRRT) are generally mild, renal toxicity has been observed, especially with highly energetic β-emitters [20]. In fact, peptides are mainly excreted through the urinary tract and in the renal proximal tubules they are reabsorbed, causing a significant radiation dose to the kidneys [21]. The co-infusion of amino acids and the administration of plasma expanders during therapy are efficient in reducing renal reabsorption but are also associated with adverse effects such as nausea and vomiting or with severe allergic reactions [22]. Other strategies aiming to reduce the absorbed dose to the kidneys and/or increase the kidney’s radiation tolerance, such as the incorporation of a cleavable linker between the targeting ligand and the radiocomplex, are under evaluation to broaden the tolerability to PRRT and enlarge its therapeutic window [23,24].

Pretargeting strategies have been extensively used in radioimmunotherapy (RIT) to improve the slow pharmacokinetics of monoclonal antibodies and optimize the delivery of their radioactive payloads [25,26,27]. This is achieved through successive injections of the targeting biomolecule and the radionuclide, with subsequent on-site and in vivo combinations. When compared to conventional targeted therapies, pretargeting allows the achievement of higher tumor/nontumor ratios, improved diagnostic and predictive properties, and minimal nontargeted collateral damage. The overall radiation exposure of the patients is reduced along with the circulation time of radioactivity, rescuing even the possibility of using short-lived radionuclides normally incompatible with antibody-based vectors [28,29]. Nonetheless, the mild conditions in which click reactions occur can also be exploited to circumvent harsh radiolabeling procedures generally required to achieve adequate radiochemical yields that would result in the degradation of the antibody and the loss of its binding affinity.

**Figure 1:**
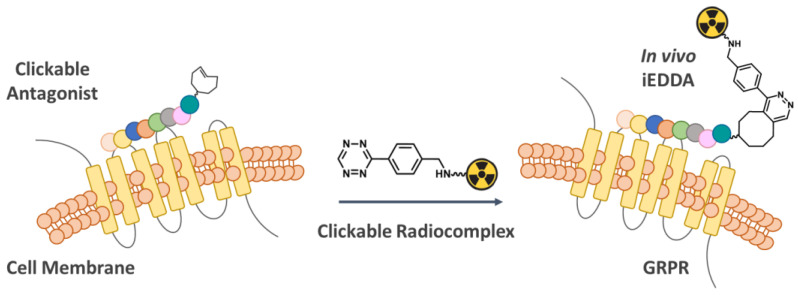
Pretargeting approach to the GRPR using clickable bombesin antagonists and ^111^In-radiocomplexes.

Since the GRPR-antagonist complex is not internalized after the binding, GRPR antagonists offer a unique opportunity to explore the pretargeting strategy using clickable bombesin antagonists for prostate cancer theranostics. Inspired by the encouraging results reported for antibodies and their fragments, the main goal of the work here described was to show that the pretargeting approach might improve the in vivo performance of GRPR-targeting peptides, namely by reducing the associated nephrotoxicity and nontargeted effects in other critical organs such as the pancreas. Toward this goal, we used the small and hydrophilic tetrazine-containing radiocomplexes previously evaluated by our group [30]. These clickable DOTA-like radiocomplexes are in fact quickly excreted through the kidneys, reducing the overall radiation exposure since the radiopeptides are formed only at the tumor site. The inverse electron-demand Diels–Alder (iEDDA) reaction between tetrazines (Tz) and trans-cyclooctenes (TCO) is extremely fast in biological media and belongs to the family of copper-free click reactions, as shown in Figure 1 [31]. One of its main advantages is the in vivo highly specific reactivity between the two chemical companions and the concomitant lack of cross-reactivity, even in a complex biological environment.

In the present study, we report on the development of clickable GRPR antagonists based on the amino acid sequence of a potent antagonist reported in several studies, D-Phe-Gln-Trp-Ala-Val-Gly-His-Sta-Leu-NH_2_ [32,33]. In addition, following the relevancy of N-terminal modifications on the receptor binding affinity and pharmacokinetic behavior, we used two different linkers to functionalize the peptide with a clickable TCO moiety [34,35]. The derivative AR-Pip-TCO was obtained upon functionalization with the short, rigid and protonable piperidine linker, while the derivative AR-PEG_4_-TCO was functionalized using the longer, flexible and neutral pegylated linker. These newly synthesized clickable antagonists were then reacted with the DOTA and DOTAGA clickable ^111^In-radiocomplexes to afford the four corresponding ^111^In-radioconjugates. These radioconjugates underwent an extensive preclinical evaluation aiming at the identification of the most promising derivative in terms of binding affinity and pharmacokinetics, to finally study the advantages of the devised pretargeting approach in vivo using GRPR antagonists.

## 2. Materials and Methods

All chemicals were purchased from Sigma-Aldrich and used as received unless otherwise specified. Rink Amide resin, HATU, HBTU and all Fmoc-protected amino acids were purchased from Novabiochem by Merck KGaA (Darmstadt, Germany). DOTA- NHS and DOTAGA anhydride were purchased from Chematech (Dijon, France). InCl_3_ (anhydrous 99%) was acquired from Alfa Aesar (Karlsruhe, Germany). [^111^In]InCl_3_ (370 MBq/mL in HCl) was obtained from Mallinckrodt (Curium) Medical B.V. (Petten, Netherlands).

### 2.1. Chemical Synthesis

The chemical synthesis of the clickable DOTA-based ligands and their radiolabeling with ^111^In have been previously reported. The chemical synthesis of the peptide AR and the preparation of the derivative AR-PEG_4_-TCO have also been previously described [30].

#### 2.1.1. Synthesis of the Clickable Bombesin Antagonist AR-Pip-TCO

AR-Pip-NH-Fmoc: The resin containing the AR peptide (25 mg of resin, approximately 8 mg, 7.2 µmol of AR peptide) was left swelling for 10 min with DCM (2 mL) before the reaction. After removal of the DCM, a solution containing Fmoc-4-amino-1-carboxymethyl-piperidine (8 mg, 3 eq.), HBTU (8 mg, 3 eq.) and DIPEA (200 µL) in 4 mL of DMF was added. The reaction was left stirring overnight at room temperature. The formation of the desired compound was confirmed by ESI-MS analysis of a small aliquot of the peptide cleaved from the resin. ESI-MS: C77H102N16O14 [M + H]+ calculated 1474.8, found 1476.0. AR-Pip-NH_2_: The removal of the Fmoc-protecting group was achieved by treating the derivative AR-Pip-NH-Fmoc attached to the resin with a mixture of 20% piperidine in DMF (*v*/*v*, total volume 3 mL) for 2 h at room temperature. The formation of the desired compound was confirmed by ESI-MS analysis of a small aliquot. The final product was cleaved from the resin using a mixture of TFA/Me_3_Si/H_2_O (2 mL/50 µL/50 µL) for 1 h at room temperature. The solution was concentrated under nitrogen to 5% of the initial volume and the desired AR-Pip-NH_2_ product was precipitated by the addition of ice-cooled Et_2_O (10 volumes). The precipitate was recovered upon centrifugation, purified by HPLC (Rt = 15.8 min) and identified by ESI-MS analysis with an overall yield over two steps of 32%. ESI-MS: C62H92N16O12 [M + H]+ calculated 1253.7, found 1254.1. AR-Pip-TCO: The compound AR-Pip-NH_2_ (2.6 mg, 2.1 µmol) was reacted with the TCO-NHS derivative (1.6 mg, 3 eq.) in the presence of 250 µL of DIPEA and 3 mL of dry DMF. After 3 h, the reaction was stopped, the solvent was removed under reduced pressure and the compound was purified by HPLC (Rt = 16.8 min), and lyophilized and characterized by ESI-MS to afford the final peptide in 34% yield. ESI-MS: C71H104N16O14 [M + H]+ calculated 1405.8, found 1405.8.

#### 2.1.2. Analytical Characterization

HPLC analysis of the described compounds (cold and radioactive) was performed using the same chromatographic equipment, a Perkin-Elmer LC 200 analytical HPLC coupled to an LC 290 UV/Vis detector and a Berthold LB-507 A radiometric detector. The column used was a Supelco Discovery BIO WidePore C18 (250 × 4.6 mm, 5 μm particle size). Mobile Phases A: H_2_O (TFA 0.1%) B: ACN (TFA 0.1%). Gradient program: 5 min A-95%, 20 min going from A-95% to B-100%, 1 min going back to A-95% and 4 min A-95%. Mass spectra were acquired in an electrospray ionization/quadrupole ion trap (ESI/QITMS) Bruker HCT mass spectrometer. Samples were injected in mixtures of H_2_O:ACN at a flow rate of 150 µL h^−1^.

### 2.2. Radiochemistry

#### 2.2.1. Radiolabeling with [^111^In]InCl_3_

The clickable radiocomplexes [^111^In]In-DOTA-Tz and [^111^In]In-DOTAGA-Tz were reacted with the peptides AR-PEG_4_-TCO and AR-Pip-TCO to afford the corresponding four radioconjugates with activities comprised between 5–20 MBq. The formation of the radioconjugates by click reaction was monitored by HPLC analysis (γ-detection) and proceeded to completion with the disappearance of the peaks corresponding to the free radiocomplexes. The retention times of the final radiolabeled peptides are reported in Table 1. The radioconjugates were purified by HPLC to remove the excess of the unreacted cold peptide that could compete for the GRPR in preclinical studies.

#### 2.2.2. Radiochemical Stability

The in vitro stability of the radioconjugates was studied by the HPLC analysis of aliquots (typically 1 to 5 MBq) of the radiolabeled compound dissolved into 4 times the volume of the desired medium PBS, cell culture media and human serum, as previously described [30].

#### 2.2.3. Lipophilicity Determination

The octanol–water partition coefficients (Po/w) of the radioconjugates (20 µL, typically 1 to 5 MBq) were determined by the “shake-flask” method, as previously described [30,36].

### 2.3. In Vitro Studies

#### 2.3.1. Cell Culture

PC3 (human prostate cancer) cells were grown in 25 cm^2^ flasks in RPMI 1640 medium, with 10% FBS and 1% Penicillin/Streptomycin. The cells were incubated at 37 °C in a saturated atmosphere of 5% CO_2_. The passage was carried out using 0.25% trypsin-EDTA after obtaining 80% confluence.

#### 2.3.2. Cellular Uptake, Internalization and Blocking Assays

The time-dependent accumulation of ^111^In-conjugates in PC3 cells was evaluated according to a previously described procedure [37]. Briefly, PC3 cells were incubated at 37 °C for 5 min to 4 h with about 7.4 kBq of the tested radioconjugates. To assess the surface binding, the cells were treated twice with glycine buffer (50 mM glycine-HCl/100 mM NaCl, pH 2.8). Then, the cells were lysed with NaOH 1 M (internalized radioconjugate). The activity in both cell surface-bound and internalized fractions was measured in a γ-counter and reported as a percentage of the total applied radioactivity. The total cellular uptake was calculated by the addition of the activities recovered from the surface binding and internalized fractions. Assays for each time point were performed in quadruplicate, and data were presented as average ± SD of typically three independent experiments.

To assess the specific GRPR-mediated cellular uptake and internalization of radioconjugates (receptor blocking studies), a similar study was performed in which cells were incubated with the [^111^In]In-DOTA-Tz-TCO-PEG_4_-AR for 1, 2 and 4 h, with or without the GRPR agonist Tyr4-Bombesin (0.25 µg/0.5 mL/well).

#### 2.3.3. Competitive Radioligand Binding Assay

The in vitro cell-binding assays were performed with the prostate carcinoma cell line PC3, as previously reported [37]. Briefly, the competition was conducted by the incubation of [^125^I]I-Tyr4-BBN in the presence of increasing concentrations (10^−16^ to 10^−5^ M) of Tyr4-BBN or the four ^nat^In-conjugates for 90 min at 4 °C. The competition was interrupted by discarding supernatants and washing the cells twice with ice-cold PBS with 0.2% of BSA. Cells were then lysed with 1 M NaOH treatment and lysates were collected and counted for their radioactivity content in an automated γ–counter (HIDEX AMG, Hidex, Turku, Finland). IC50 values were calculated by nonlinear regression according to one-site or two-site models using GraphPad Prism 5 software (San Diego, CA, USA) and are the average of three independent experiments.

### 2.4. Animal Studies

The animal studies were performed according to national and EU legislation for good practices on animal care, protection and welfare.

The biodistribution and the ability of ^111^In-radioconjugates to be retained in tumors were evaluated in Balb/c-male nude mice with PC3 xenografts as previously reported [30]. Briefly, xenografts were induced by the subcutaneous injection of 8 × 10^6^ PC3 cells in Matrigel:PBS buffer 1:1 in the right flank of the mice. When palpable tumors were developed, animals were injected intravenously with 100 µL (1.6–5.8 MBq/44–150 μCi) of each ^111^In-radioconjugate. Mice were sacrificed at 15 min, 1 h, 2 h and 24 h post-injection. The injected radioactive dose and the radioactivity remaining in the animal after sacrifice were measured in a dose calibrator (Capintec CRC25R). Blood samples were taken by cardiac puncture at sacrifice. The main organs were dissected and weighted, and their radioactivity was measured using a gamma counter (HIDEX AMG, Hidex, Turku, Finland). The uptake in the tissues was calculated as a percentage of the injected dose per gram of tissue (%I.D./g). Statistical analysis (Paired *t*-test) was performed using GraphPad Prism 5 software (San Diego, CA, USA).

To assess the pretargeted approach, the same animal model was previously injected in the tail vein with 1 nmol of the TCO-containing GRPR antagonist AR-PEG_4_-TCO followed by the injection of [^111^In]In-DOTA-Tz after an interval of 4 h. At selected time points, the animals were sacrificed and the biodistribution was assessed using the same experimental procedure.

## 3. Results

### 3.1. Synthesis of the TCO-Clickable GRPR Antagonists

The potent GRPR antagonist with sequence D-Phe-Gln-Trp-Ala-Val-Gly-His-Sta-Leu-NH_2_ (that we will refer to as AR) was synthesized by solid-phase peptide synthesis (SPPS) using an automated synthesizer. After cleavage from the resin, the peptide was purified using HPLC analysis and identified by ESI-MS.

The clickable antagonist AR-PEG_4_-TCO was obtained by reacting the AR peptide with TCO-PEG_4_-NHS in 57% yields. The final product was identified by ESI-MS analysis, purified using HPLC and lyophilized. The derivative AR-Pip-TCO was obtained by reacting first the AR peptide with the Fmoc-protected piperidine linker. Then, the protecting group was removed and the free amino group was coupled to the TCO-NHS derivative. The desired final product was obtained in a final 13% yield (calculated over three steps) and was identified by ESI-MS analysis, purified using HPLC and lyophilized.

### 3.2. Synthesis of the ^111^In-Radioconjugates

The [^111^In]In-DOTA-Tz-TCO-PEG_4_-AR, [^111^In]In-DOTAGA-Tz-TCO-PEG_4_-AR, [^111^In]In DOTA-Tz-TCO-Pip-AR and [^111^In]In-DOTAGA-Tz-TCO-Pip-AR were synthesized to perform preliminary in vitro studies and identify the most promising compound to test our GRPR pretargeting strategy.

**Figure 2:**
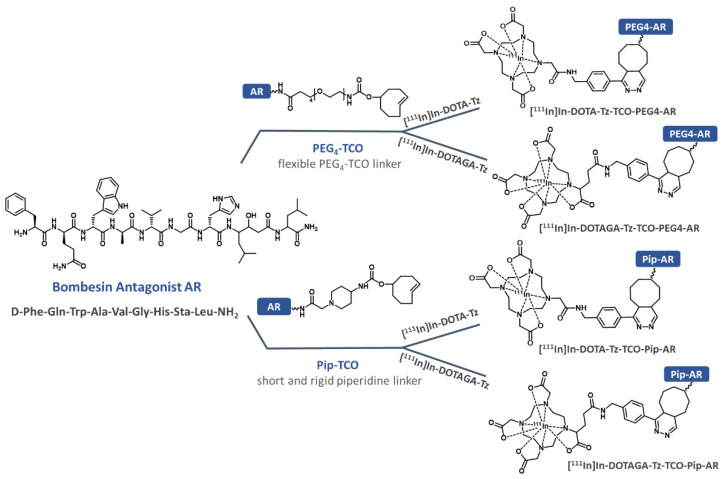
Synthesis of the ^111^In-radioconjugates by click reaction between the clickable antagonists AR-PEG_4_-TCO and AR-Pip-TCO and the clickable radiocomplexes [^111^In]In-DOTA-Tz and [^111^In]In -DOTAGA-Tz.

The ^111^In-radioconjugates shown in Figure 2 were obtained by an indirect radiolabeling strategy upon the iEDDA click reaction between the newly synthesized clickable GRPR antagonists AR-PEG_4_-TCO and AR-Pip-TCO and the radiocomplexes [^111^In]In-DOTA-Tz and [^111^In]In-DOTAGA-Tz.

**Figure 3:**
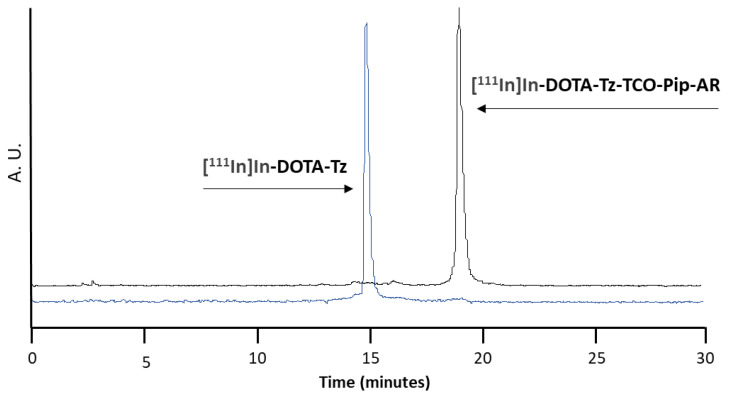
HPLC chromatograms (γ-detection) of [^111^In]In-DOTA-Tz in blue (Rt = 14.8 min) and ^111^In-DOTA-Tz-TCO-Pip-AR in black (Rt = 19.1 min).

The click reaction was performed in an aqueous solution at 37 °C. After 1 h, the HPLC analysis (γ-detection) of the mixture showed the disappearance of the peak corresponding to the clickable DOTA-like radiocomplex, indicating the completion of the reaction, as shown in Figure 3.

The chemical identity of the newly formed radioconjugates was corroborated by the injection of the cold ^nat^In-conjugates with identical retention times (see Figure S1 in the Supplementary Materials), prepared using the ^nat^In-DOTA and ^nat^In-DOTAGA complexes. The synthesis of both ^nat^In and ^111^In complexes have been previously described [30]. The formation of a smaller split peak corresponding to the dihydropyridazine isomers was also observed (see Figure S2, Supplementary Materials), but slowly disappeared as it was oxidizing into the corresponding final aromatic construct [38].

Several antagonists bearing a combination of the same amino acid sequence, chelators and linkers were previously reported, however, to the best of our knowledge, it is the first time that those conjugates were obtained through a click reaction and a Tz-TCO motif is incorporated within the structure. Therefore, the following studies aimed at the evaluation of these chemical modifications, not only on their radiochemical stability but also, and most importantly, on their binding affinity toward the GRPR.

### 3.3. In Vitro Studies

The radiochemical stability of the radioconjugates was evaluated in vitro by the incubation of the four radioconjugates at 37 °C in PBS, in cell culture media (CCM) and human serum (HS) for 24 h, followed by HPLC analysis. All radioconjugates exhibited excellent stabilities in PBS and CCM (above 95%), with no degradation in up to 24 h of incubation (see Figure S3 in the Supplementary Materials). In HS, the derivatives bearing a DOTA moiety demonstrated very good radiochemical stability, while the derivative [^111^In]In-DOTAGA-Tz-TCO-PEG_4_-AR had the lowest stability value, around 70%. Nonetheless, the excellent radiochemical stability of all radioconjugates in CCM was particularly relevant for the following studies aimed at the evaluation of their GRPR binding affinity using a prostate cancer in vitro model.

The lipophilicity of the radioconjugates was evaluated by the determination of the partition coefficient log Po/w, using the shake-flask method; the results are shown in Table 1.

The values of log Po/w of the radioconjugates indicate that all compounds are quite hydrophilic. The derivatives bearing the pegylated spacer, in the first two entries of Table 2, had almost the same log Po/w value of −1.2. Surprisingly, the negative charge of the [In(III)-DOTAGA]^−1^ moiety did not appear to influence the hydrophilicity of the final compound.

The radioconjugate [^111^In]In-DOTA-Tz-TCO-Pip-AR showed a more negative log Po/w value of −1.28, suggesting that the introduction of the protonable piperidinyl linker might contribute to a higher extent to the overall hydrophilicity of this DOTA-based compound. On the other hand, the derivative [^111^In]In-DOTAGA-Tz-TCO-Pip-AR is the least hydrophilic, with a log Po/w value of −0.9 that might be explained by a compensation between the two opposite charges from the complex and the linker at physiological pH.

### 3.4. Cellular Studies

The radioconjugates [^111^In]In-DOTA-Tz-TCO-PEG_4_-AR, [^111^In]In-DOTAGA-Tz-TCO-PEG_4_-AR, [^111^In]In-DOTA-Tz-TCO-Pip-AR and [^111^In]In-DOTAGA-Tz-TCO-Pip-AR were studied to determine their uptake and internalization in a prostate cancer in vitro model using the well-established GRPR-positive cell line, PC3.

**Figure 4:**
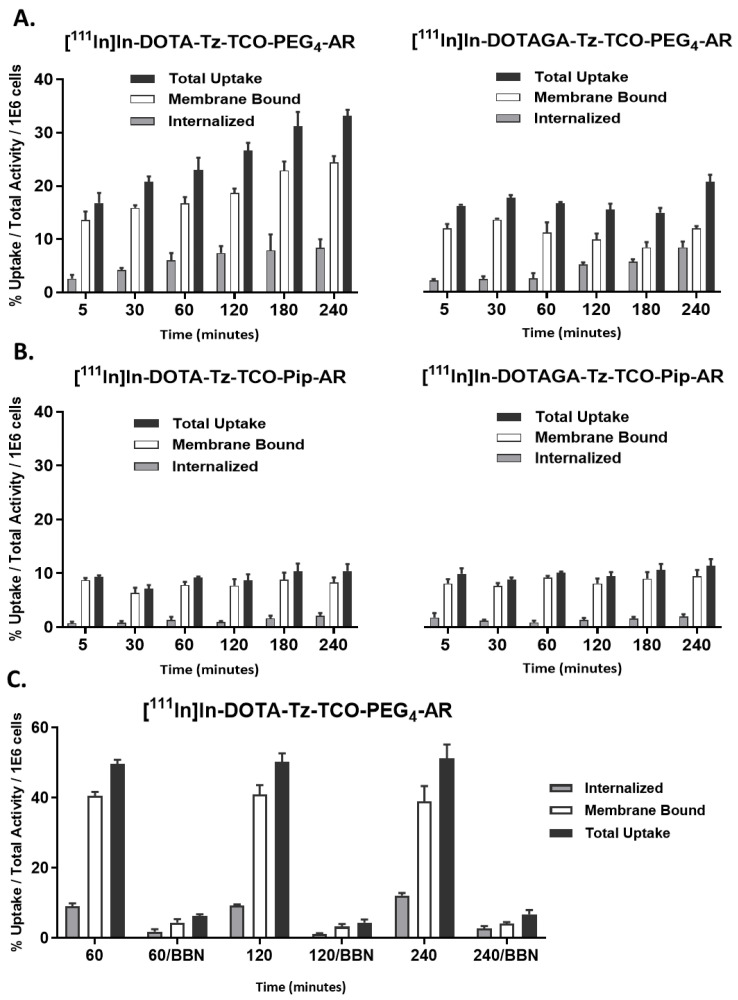
Time-dependent cellular uptake, surface-bound and internalization of the radioconjugates in PC3 cells at 37 °C. (**A**). [^111^In]In-DOTA-Tz-TCO-PEG_4_-AR and [^111^In]In-DOTAGA-Tz-TCO-PEG_4_-AR. (**B**). [^111^In]In-DOTA-Tz-TCO-Pip-AR and [^111^In]In-DOTAGA-Tz-TCO-Pip-AR. (**C**). [^111^In]In-DOTA-Tz-TCO-PEG4-AR co-incubated with or without the GRPR agonist Tyr4-Bombesin (0.25 µg/0.5 mL/well). The results are expressed as a percentage of the total (applied) activity (mean ± SD; *n* = 4).

Despite bearing the same carrier peptide, the four derivatives showed biological behaviors influenced by both the linker and the chelator moiety inserted into the structure. The derivatives carrying the longer and flexible pegylated linker showed higher cellular uptake values and the derivatives bearing the DOTAGA chelator demonstrated lower cellular uptake than the DOTA congeners, as shown in Figure 4A. On the other hand, the derivative [^111^In]In-DOTA-Tz-TCO-PEG_4_-AR had the highest cellular uptake at around 35% of the total activity applied per million cells after 4 h of incubation, eventually reflecting its highest affinity for the GRPR. Furthermore, this derivative was the only one showing a slow but steady increase in uptake over time, doubling the uptake value (17% to 33% of the total activity applied per million cells) at the end of the assay. Most importantly, the derivative [^111^In]In-DOTA-Tz-TCO-PEG4-AR was mainly bound to the cell membrane, which is consistent with antagonist behavior.

The radioconjugates [^111^In]In-DOTA-Tz-TCO-Pip-AR and [^111^In]In-DOTAGA-Tz-TCO-Pip-AR, bearing the protonable piperidinyl linker, showed moderate uptake values with a maximum uptake of around 10% of the total activity applied per million cells. The trend of the uptake and internalization was similar for both conjugates, without significative enhancements over time, as shown in Figure 4B. Nevertheless, almost all cell-associated activity corresponded to the membrane-bound fraction, confirming their antagonist behavior.

The specificity of the uptake of the radioconjugate [^111^In]In-DOTA-Tz-TCO-PEG_4_-AR was confirmed by repeating the assay in the presence of an excess of the GRPR agonist bombesin (Tyr4-Bombesin). The addition of the agonist successfully saturated the GRPR binding sites and prevented the uptake of the radioconjugate, as shown in Figure 4C. An average percentage of inhibition of 88%, constant over the whole assay, was observed.

**Figure 5:**
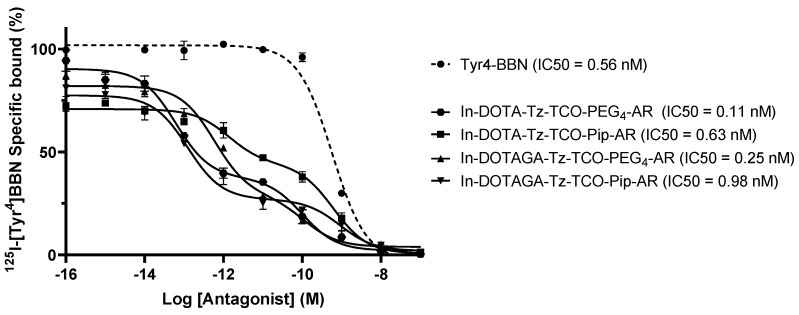
Displacement curves of [^125^I]I-Tyr4-bombesin by the Tyr4-BBN and the four ^nat^In-conjugates with respective IC50 values (corresponding to the lower affinity binding site).

We next evaluated the GRPR binding affinity of the four derivatives obtained by the complexation of the DOTA and DOTAGA ligands with ^nat^In and the following conjugation with the peptides AR-PEG_4_-TCO and AR-Pip-TCO. The binding curves obtained suggested the presence of two binding sites, as shown in Figure 5. Through a two-site nonlinear regression analysis, the IC50 values for the lower affinity binding sites (IC50Lo) were calculated and found to be in the nanomolar range, in agreement with the values reported in the literature for the reference peptide AR (2.9 ± 0.6 nM) and several GRPR antagonists with a similar structure [32,39].

The existence of a second GRPR binding site (see Table S1) with considerably higher affinity has never been described before for PC3 human prostate cancer cells, despite some examples which have been reported in the literature for bombesin/GRP binding in human breast cancer cells and mouse colon cancer cells [40,41]. Nonetheless, the results obtained allowed us to conclude that the chemical modifications of the peptides with the TCO-Tz moieties did not negatively affect the binding affinity and resulted in metallated conjugates with very good IC_50_ nanomolar values.

The derivatives bearing the pegylated linker showed higher binding affinities than those carrying the Pip linker (0.11 versus 0.63 nM for the DOTA-based and 0.63 versus 0.98 nM for the DOTAGA-based conjugates, respectively), in agreement with the results obtained in the cellular uptake assays.

### 3.5. In Vivo Studies

We next extended the preclinical evaluation of the GRPR antagonists to a prostate cancer in vivo model, using male Balb/C nude mice bearing PC3 xenografts. For such purpose, we selected the derivative [^111^In]In-DOTA-Tz-TCO-PEG_4_-AR that displayed the highest cellular uptake and GRPR binding affinity in the cellular studies, and we compared the biodistribution profile with the corresponding DOTA-based radiopeptide bearing the piperidinyl linker.

**Figure 6:**
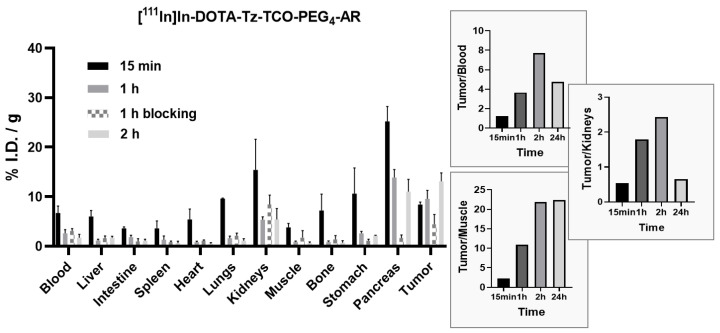
Biodistribution data of [^111^In]In-DOTA-Tz-TCO-PEG_4_-AR (including tumor/nontarget organs ratios in the insert) in PC3 xenografts-bearing mice, expressed as %I.D./g of organ (mean ± SD, *n* = 3).

[^111^In]In-DOTA-Tz-TCO-PEG_4_-AR demonstrated good and fast uptake values in the tumor and in the tissues expressing the GRPR receptor such as the pancreas (8.4 ± 0.5 and 25.2 ± 3%I.D./g, respectively, at 15 min post-injection), as shown in Figure 6. At one-hour post-injection, the uptake was still high with 9.6 ± 1.7%I.D./g in the tumor and 13.9 ± 1.6%I.D./g in the pancreas. At the same time point, blocking of the receptor with an excess of agonist successfully hindered the pancreas and tumor uptake, which were reduced to 1.6 ± 0.6 and 4.4 ± 2%I.D./g, respectively. At two hours post-injection, the uptake in the pancreas and tumor remained constant, with uptake values of 11 ± 2.5 and 13.1 ± 1.7, respectively. At 24 h post-injection, 97% of the injected activity was excreted, being mainly eliminated through the renal system.

In contrast, the derivative [^111^In]In-DOTA-Tz-TCO-Pip-AR bearing the piperidinyl linker (see Figure S4 and Table S3 in the Supplementary Materials) showed lower uptake values in the tumor and pancreas, 3.2 ± 0.1 and 2.8 ± 0.5%I.D./g, respectively, at one-hour post-injection. The blockade of the receptor at the same time point successfully reduced the uptake. Taken together, these results indicate that the compound [^111^In]In-DOTA-Tz-TCO-PEG_4_-AR is able to specifically target the GRPR in vivo with a better in vivo performance than the congener carrying the Pip linker, and is therefore a suitable candidate for the implementation of a pretargeted approach to GRPR.

**Figure 7:**
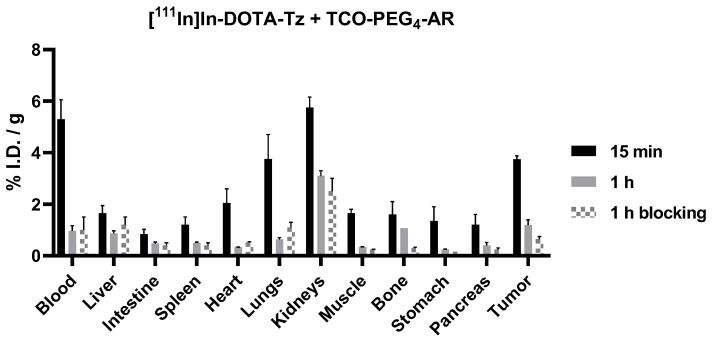
Biodistribution data of the pretargeted approach based on the administration of 50 µg of the AR-PEG_4_-TCO peptide, followed by a 4 h later injection of the clickable radiocomplex [^111^In]In-DOTA-Tz in PC3 xenografts-bearing mice, expressed as %I.D./g of organ (mean ± SD, *n* = 3).

Consequently, we injected the TCO-containing GRPR antagonist AR-PEG_4_-TCO in Balb/C nude mice bearing a PC3 xenograft, and after an interval of 4 h, we injected the small clickable radiocomplex [^111^In]In-DOTA-Tz. As a term of comparison, we also studied the biodistribution of the Tz-containing clickable radiocomplex alone, and the results are presented in Figure 7 and Tables S4 and S5 in the Supplementary Materials. For the pretargeting approach, a good tumor uptake value was observed at 15 min post-injection but the activity was quickly washed out at 1 h post-injection (3.7 ± 0.1 and 1.2 ± 0.2%I.D./g, respectively). Nonetheless, considerably lower tumor uptake was observed when comparing the results of the classical and pretargeted approaches based on the administration or in vivo formation of [^111^In]In-DOTA-Tz-TCO-PEG_4_-AR (8.4 ± 0.5 versus 3.7 ± 0.1%I.D./g at 15 min post-injection, respectively).

The results obtained in the pretargeting experiment indicated a higher uptake in the tumor and pancreas when compared with the results obtained upon injection of the clickable [^111^In]In-DOTA-Tz complex alone: (i) at 15 min post-injection, 3.7 ± 0.1 versus 2.46 ± 0.6%I.D./g and 1.2 ± 0.4 versus 0.42 ± 0.02%I.D./g, respectively; and (ii) at 1 h post-injection, 1.19 ± 0.2 versus 0.5 ± 0.2%I.D./g and 0.4 ± 0.11 versus 0.09 ± 0.06%I.D./g, respectively. Despite the modest increase, the results are significant, as confirmed by the paired *t*-test value (*p*-value = 0.0313). Furthermore, in the pretargeting experiment, the tumor and pancreas uptake at 1 h post-injection were reduced upon GRPR blockade with a BBN agonist, from 1.19 ± 0.2 to 0.64 ± 0.1%I.D./g and from 0.4 ± 0.11 to 0.25 ± 0.05%I.D./g, respectively.

Concerning the nontarget tissues, in GRPR-targeted radionuclide therapy, the main nontarget organs at risk are the kidneys because of the tubular reabsorption and the pancreas because of the high GRPR expression [42,43]. The pretargeted approach allowed the lowering of the dose to the kidneys with uptake values of 5.75 ± 0.4 versus 15.4 ± 6.2%I.D./g at 15 min p.i. and 3.1 ± 0.2 versus 5.3 ± 0.6%I.D./g at 1 h p.i. In the pancreas, the activity passed from 25.2 ± 0.2 to 1.2 ± 0.4%I.D./g and from 13.9 ± 1.6 to 0.4 ± 0.11%I.D./g at 15 min and 1 h post-injection, respectively.

## 4. Discussion

The study hereby presented allowed the preclinical evaluation of four radioconjugates obtained upon the click reaction between a TCO-functionalized GRPR antagonist and a Tz-containing ^111^In-radiocomplex. The clickable GRPR antagonists were prepared by attaching the TCO-clickable moiety through two different linkers, one more flexible and composed of four ethylene glycol units and another, shorter and less flexible, based on a piperidinyl ring. The resulting derivatives AR-PEG_4_-TCO and AR-Pip-TCO were then used to obtain four radioconjugates by click reaction with the radiocomplexes [^111^In]In-DOTA-Tz and [^111^In]In-DOTAGA-Tz already described in previous work of our group. The synthesis of the radioconjugates by click reaction was performed in water at 37 ˚C and was complete after 1 h of incubation, as corroborated by the disappearance of the peak corresponding to the radiocomplexes in the HPLC. The final four radioconjugates demonstrated excellent in vitro radiochemical stabilities.

The four radioconjugates were then evaluated in several cellular studies demonstrating good and selective GRPR uptake and excellent binding affinities in the low nanomolar range, especially for the derivative [^111^In]In-DOTA-Tz-TCO-PEG_4_-AR. Biodistribution studies with this derivative have further confirmed the promising cellular results with a good and persistent uptake in the tumor and the GRPR-expressing tissues.

Based on these encouraging results, we have proceeded to the application of a pretargeted approach to GRPR-expressing tumors, which is almost unexplored. Click chemistry has previously been applied to bombesin derivatives but mainly to improve metabolic stability or to achieve convenient ^18^F-radiolabeling [44,45,46]. However, such reactions based on triazole formation and catalyzed by copper are not suitable for pretargeting in vivo applications. The rationale for applying this approach, commonly used for biomolecules with slow pharmacokinetics such as monoclonal antibodies, was dual. On one side, improving the peptide pharmacokinetic aiming to reduce the kidney toxicity that is often observed in PRRT and the reduction in pancreatic off-target effects commonly found in PRRT using radiolabeled peptides toward GRPR. On the other hand, we also sought the versatility of the DOTA-based radiocomplexes that allow quantitative radiolabeling with several theranostic isotopes (e.g., ^68^Ga/^177^Lu and ^64^Cu/^67^Cu) and possibly obtain the corresponding radioconjugates by a fast and mild click reaction for their use in a broad plethora of applications.

Our study was based on successive injections of a clickable GRPR antagonist followed by a 4 h interval and successive administration of the [^111^In]In-DOTA-Tz radiocomplex. The tumor uptake observed at 15 min post-injection was quickly washed out when compared to the biodistribution data of the classic approach. However, comparing the biodistribution data of pretargeting with the results obtained by only injecting the [^111^In]In-DOTA-Tz radiocomplex, there is an augmented and significant tumor and pancreas uptake.

The modest uptake values might be explained by a fast in vivo metabolization of the TCO-containing peptides that are shortly available after the pretargeting interval for the click reaction with the radiocomplex. When compared to monoclonal antibodies, peptides, have very short plasmatic half-lives that might hamper this approach, at least in the experimental conditions that we have described. However, the results presented are promising and demonstrate that it is worth pursuing the optimization of several parameters to improve the in vivo results.

For instance, simple measures such as the reduction in the pretargeted interval that might be too long for these types of molecules or the insertion of a moiety capable of extending the plasmatic half-life in the structure of the clickable peptides might improve the outcome of the pretargeting approach using GRPR antagonists. A previous work using a GRPR antagonist conjugated to an albumin-binding domain successfully improved the circulating time of the radioconjugate, but also highlighted a very high kidney uptake, hampering a possible use in radionuclide therapy [43]. The pretargeting approach described herein, which successfully reduced the doses delivered to the kidneys and pancreas, might be ideal to improve the biodistribution profile of these types of radioconjugates.

Nonetheless, besides the possibility of exploring the pretargeting approach, we also consider that the use of this strategy based on clickable GRPR antagonists and DOTA/DOTAGA derivatives allowing fine-tuning of their pharmacokinetics and metabolic stability might enable a more straightforward and versatile synthesis of new libraries of (radio)conjugates for the development of theranostic tools toward GRPR-expressing tumors.

## Figures and Tables

**Table 1:** Retention times (min) of the [^111^In]In-Radioconjugates.

Compound	Retention Time (min)
[^111^In]In-DOTA-Tz-TCO-PEG_4_-AR	19.8
[^111^In]In-DOTAGA-Tz-TCO-PEG_4_-AR	20.4
[^111^In]In-DOTA-Tz-TCO-Pip-AR	19.1
[^111^In]In-DOTAGA-Tz-TCO-Pip-AR	19.3

**Table 2:** Partition coefficients (log Po/w) of the four ^111^In-radioconjugates (*n* = 3 or 4).

Compound	Log Po/w
[^111^In]In-DOTA-Tz-TCO-PEG_4_-AR	−1.2 ± 0.04
[^111^In]In-DOTAGA-Tz-TCO-PEG_4_-AR	−1.18 ± 0.05
[^111^In]In-DOTA-Tz-TCO-Pip-AR	−1.28 ± 0.01
[^111^In]In-DOTAGA-Tz-TCO-Pip-AR	−0.9 ± 0.00

## Supplementary Materials

The following supporting information can be downloaded at: https://www.mdpi.com/article/10.3390/pharmaceutics14122569/s1, Figure S1. A. HPLC analysis (UV detection at 220 nm) of the TCO-bearing peptides with the PEG4 and Pip linkers, B. the corresponding four cold DOTA-based ^nat^In-conjugates, and C. the corresponding four cold DOTAGA-based ^nat^In-conjugates. Figure S2. HPLC analysis of the formation of the dihydropyridazine isomers for the compound DOTA-Tz-TCO-PEG_4_-AR (t_R_ = 14.9 min). Figure S3. Radiochemical purity (estimated by HPLC) of the radioconjugates after incubation in different media at 37 °C for 24 h. Table S1. Binding affinity expressed as IC50 values obtained by nonlinear regression analysis of the competitive binding assay (two sites). Figure S4. Biodistribution data for [^111^In]In-DOTA-Tz-TCO-Pip-AR in PC3 xenografts-bearing mice, expressed as %I.D./g of organ (mean ± SD, *n* = 3). Table S2. Biodistribution data of [^111^In]In-DOTA-Tz-TCO-PEG4-AR in PC3-xenografts-bearing mice at 15 min, 1 h (with and without blocking), 2 h and 24 h p.i. (data expressed as mean %I.D./g and SD, *n* = 3). Table S3. Biodistribution data of [^111^In]In-DOTA-Tz-TCO-Pip-AR in PC3-xenografts-bearing mice at 1 h (with and without blocking) and 2 h p.i. (data expressed as mean %I.D./g and SD, *n* = 3). Table S4. Biodistribution data of [^111^In]In-DOTA-Tz in PC3-xenografts-bearing mice at 15 min, 1 h and 24 h p.i. (data expressed as mean %I.D./g and SD, *n* = 3). Table S5. Biodistribution data of the GRPR pre-targeting approach by injection of [^111^In]In-DOTA-Tz 4h p.i. of AR-PEG4-TCO (50 g) in PC3-xenografts bearing mice at 15 min and 1 h (with and without blocking) p.i. (data expressed as mean %I.D./g and SD, *n* = 3).
