# Supplementary material for: Bioorthogonal Chemistry Approach for the Theranostics of GRPR-Expressing Cancers
Source: Pharmaceutics. 2022 Nov 23;14(12):2569. doi: 10.3390/pharmaceutics14122569 (PMC9785946; doi:10.3390/pharmaceutics14122569)
Supplement: Supplementary file 1 [file pharmaceutics-14-02569-s001.zip › pharmaceutics-2008075-supplementary.pdf]

Bioorthogonal Chemistry Approach for Theranostics of GRPR-Expressing Cancers

A. D'Onofrio [1,*], F. Silva [1,], L. Gano [1,2], P. Raposinho [1,2], C. Fernandes [1,2], A. Sikora [3], M. Orzełowska [3], R. Mikołajczak [3], P. Garnuszek [3], A. Paulo [1,2~]**

1 Centro de Ciências e Tecnologias Nucleares, Instituto Superior Técnico, Universidade de Lisboa, Campus Tecnológico e Nuclear, Estrada Nacional 10, Km 139.7, 2695-066 Bobadela LRS, Portugal

2 Departamento de Engenharia e Ciências Nucleares, Instituto Superior Técnico, Universidade de Lisboa, Portugal

3 National Centre for Nuclear Research, Radioisotope Centre POLATOM, Otwock, Poland

* Correspondence: alicedonofrio@gmail.com

** Present address: Champalimaud Centre for the Unknown, Fundação Champalimaud, Av. Brasília, 1400-038 Lisboa

Table of Contents

Figure S1. **2-4**

Figure S2. **5**

Figure S3. **5**

Table S1. **6**

Figure S4. **6**

Table S2. **7**

Table S3. **7**

Table S4. **8**

Table S5. **8**

A.

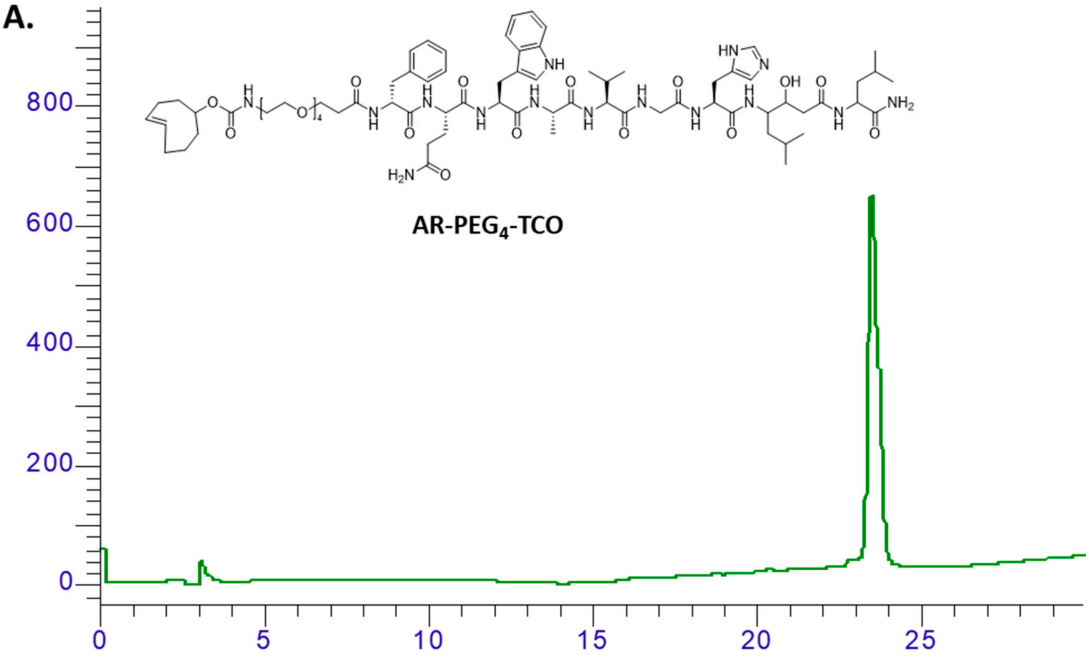
AR-PEG4-TCO
800
600
400
200
0
0
5
10
15
20
25

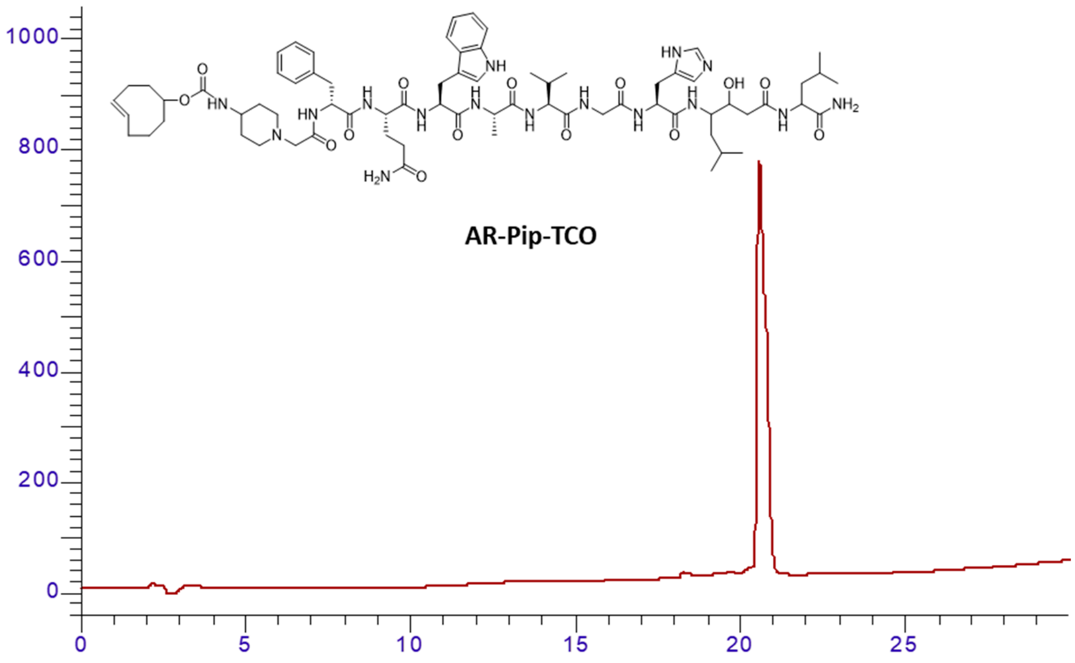
AR-Pip-TCO
1000
800
600
400
200
0
0
5
10
15
20
25

B.

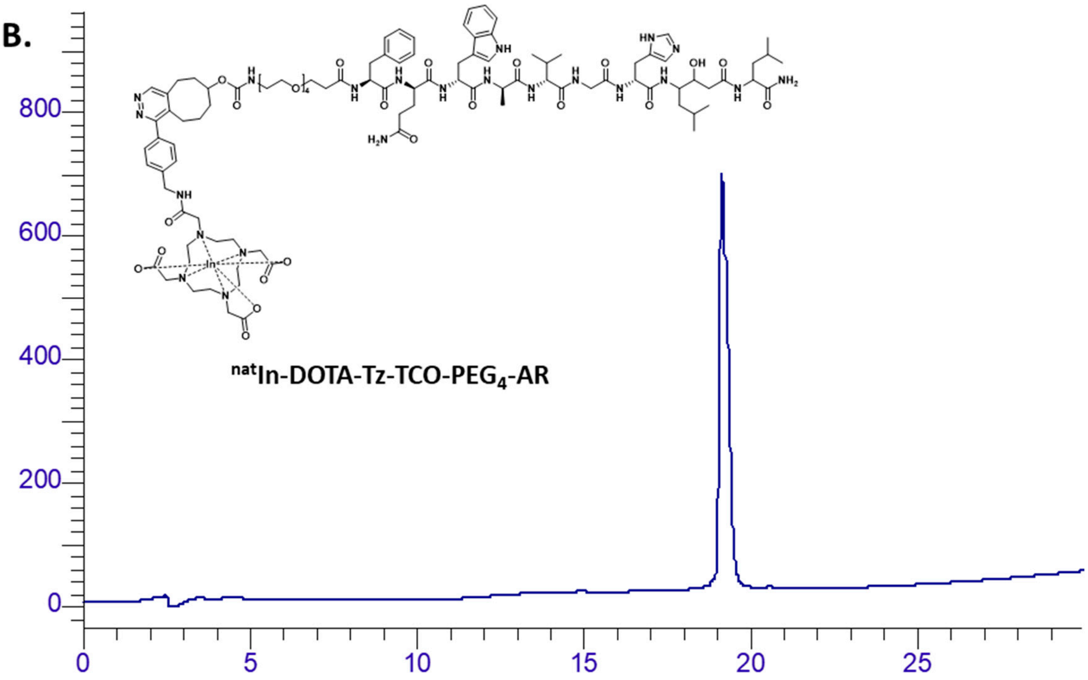

natIn-DOTA-Tz-TCO-PEG4-AR
800
600
400
200
0
0
5
10
15
20
25

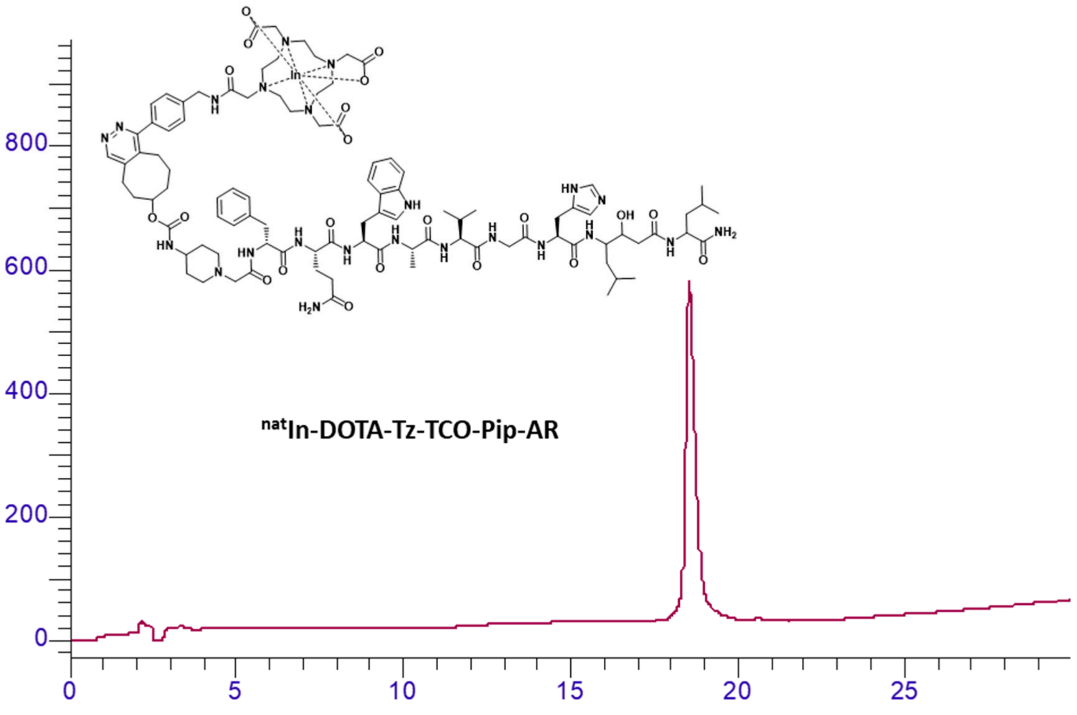

natIn-DOTA-Tz-TCO-Pip-AR
800
600
400
200
0
0
5
10
15
20
25

C.

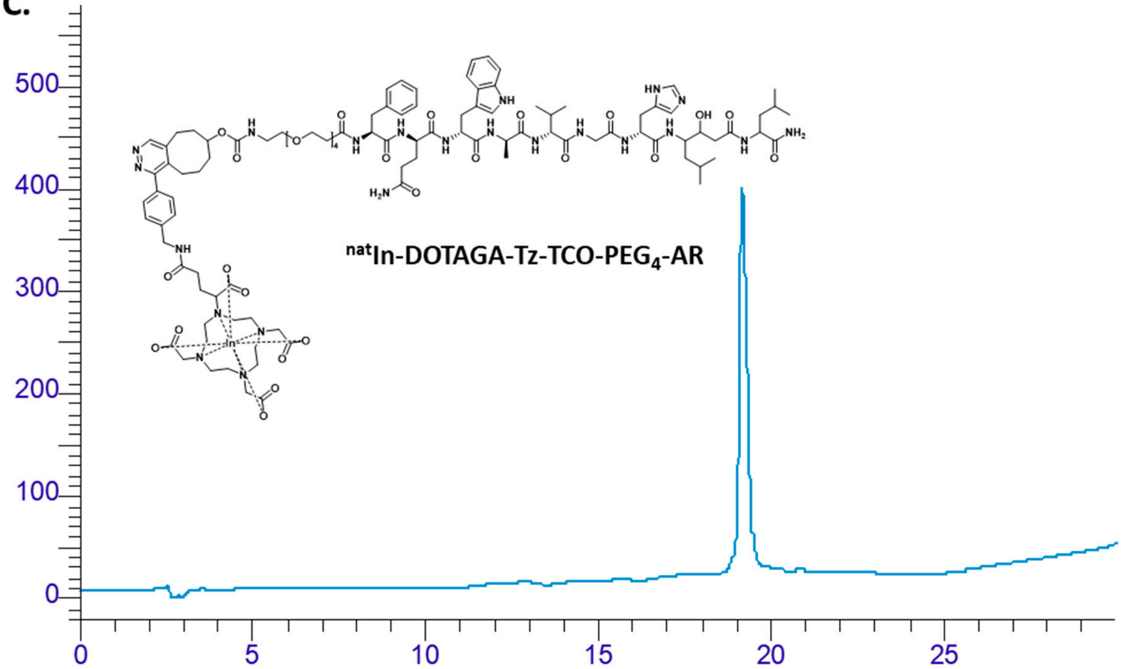

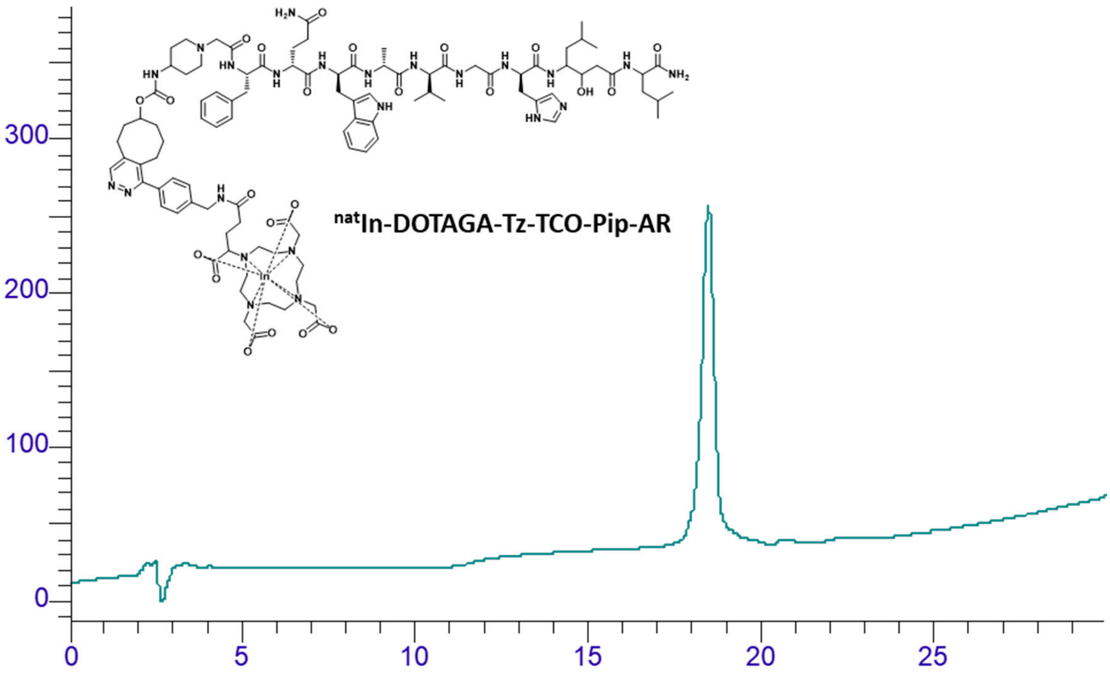

Figure S1. HPLC analysis (UV detection at 220 nm) of the **A.** TCO-bearing peptides with the PEG_4 and Pip linkers (Rt = 23.6 and 20.9 min, respectively) **B.** the corresponding cold DOTA-based ^{nat}In-conjugates (Rt = 19.1 and 18.5 min, respectively) and **C.** DOTAGA-based ^{nat}In-conjugates (Rt = 19.4 and 18.6 min, respectively).

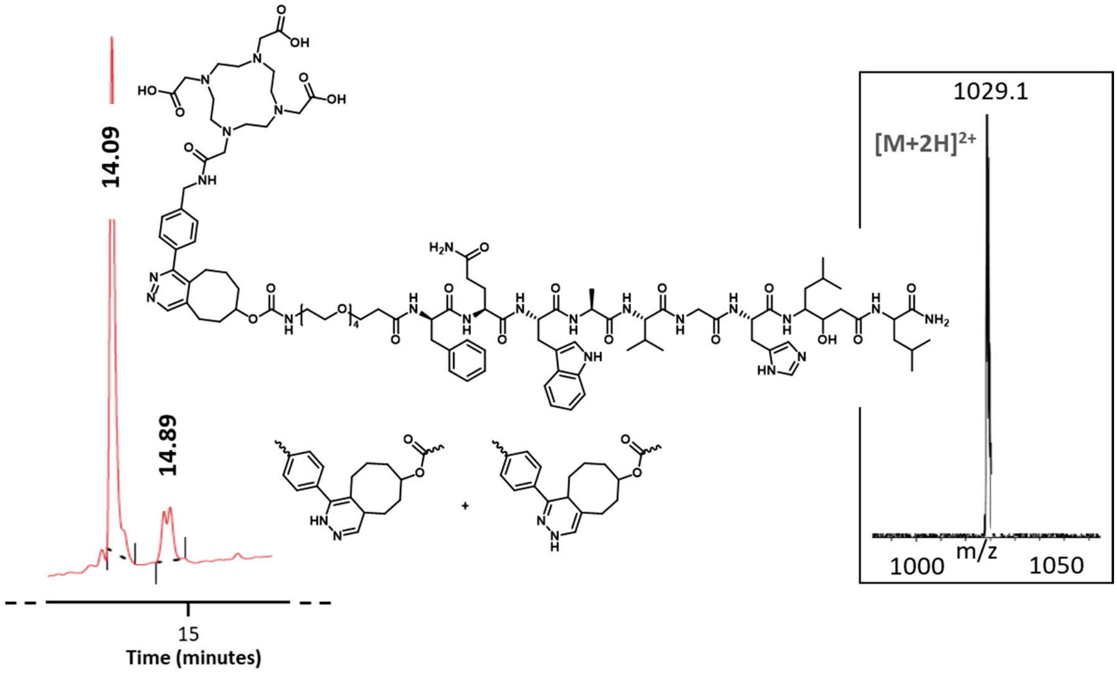

Figure S2. HPLC analysis of the formation of the dihydropyridazine isomers (t_R = 14.9 min) upon click reaction leading to the formation of the compound DOTA-Tz-TCO-PEG4-AR (t_R = 14.1 min).

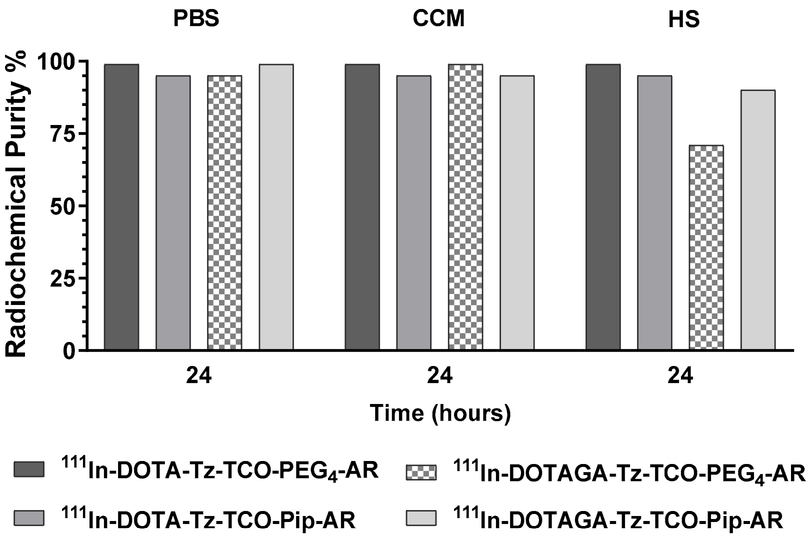

Figure S3. Radiochemical purity (estimated by HPLC) of the radioconjugates after incubation in different media (PBS, RPMI 1640 medium supplemented with 10% FBS and 1% Penicillin/Streptomycin and Human Serum) at 37°C for 24 hours.

Table S1. Binding Affinity expressed as IC50 values obtained by non-linear regression analysis of competitive binding assay (two-sites).

	In-DOTA-Tz-TCO-PEG4-AR	In-DOTA-Tz-TCO-Pip-AR	In-DOTAGA-Tz-TCO-PEG4-AR	In-DOTAGA-Tz-TCO-Pip-AR	Tyr4-BBN
**IC50Hi**	6.20E-14	1.36E-12	5.89E-13	1.23E-13	/
**IC50Lo**	1.01E-10	6.19E-10	1.13E-10	9.00E-10	5.73E-10

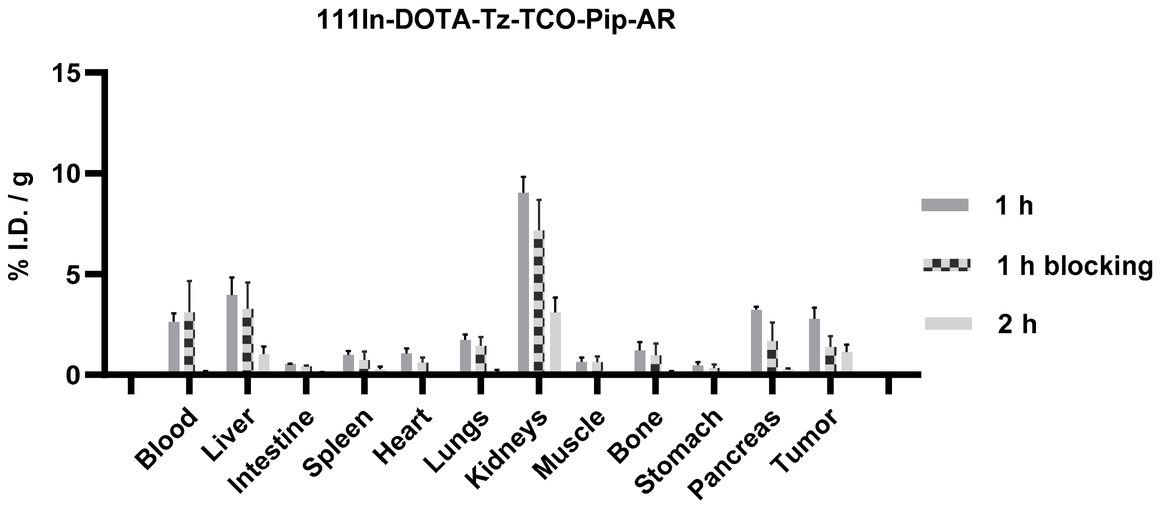

Figure S4. Biodistribution data for [111In]In-DOTA-Tz-TCO-Pip-AR in PC3 xenografts bearing mice, expressed as % I.D./g of organ (mean ± S.D., n = 3).

Table S2. Biodistribution data of [^{111}In]In-DOTA-Tz-TCO-PEG4-AR in PC3-xenografts bearing mice at 15 min, 1 h (with and without blocking), 2 h and 24 hours p.i. (data expressed as mean % I.D./g and SD, n = 3).

111In-DOTA-Tz-TCO-PEG4-AR	MEAN	SD	MEAN	SD	MEAN	SD	MEAN	SD	MEAN	SD
	15 min		1 h		1 h blocking		2 h		24 h	
Blood	6.7	*1.4*	2.62	*0.74*	3.08	*0.46*	1.7	*0.7*	0.16	*0.01*
Liver	6	*1.2*	1.15	*0.275*	1.63	*0.44*	1.7	*0.3*	0.21	*0.02*
Intestine	3.6	*0.3*	1.855	*0.365*	0.995	*0.51*	1.1	*0.3*	0.12	*0.09*
Spleen	3.6	*1.5*	1.32	*0.75*	0.83	*0.21*	0.6	*0.4*	0.065	*0.006*
Heart	5.4	*2.1*	0.815	*0.19*	1.11	*0.18*	0.5	*0.2*	0.065	*0.005*
Lungs	9.6	*0.1*	1.615	*0.43*	2.135	*0.51*	1.1	*0.4*	0.12	*0.02*
Kidneys	15.4	*6.2*	5.335	*0.625*	8.435	*1.88*	5.4	*2.2*	1.17	*0.23*
Muscle	3.8	*0.8*	0.875	*0.18*	1.755	*1.41*	0.6	*0.3*	0.034	*0.003*
Bone	7.2	*3.3*	0.85	*0.23*	1.395	*0.75*	0.7	*0.4*	0.038	*0.0014*
Stomach	10.6	*5.2*	2.625	*0.395*	1.16	*0.27*	2.1	*0.1*	0.09	*0.02*
Pancreas	25.2	*3*	13.875	*1.595*	1.685	*0.59*	11	*2.5*	0.14	*0.09*
Tumor	8.4	*0.5*	9.575	*1.705*	4.445	*1.97*	13.1	*1.7*	0.76	*0.09*
Excretion	17.4	*9.8*	74.85	*7.05*	51.85	*14.35*	84.7	*8.7*	96.8	*0.2*

Table S3. Biodistribution data of [^{111}In]In-DOTA-Tz-TCO-Pip-AR in PC3-xenografts bearing mice at 1 h (with and without blocking) and 2h p.i. (data expressed as mean % I.D./g and SD, n = 3).

111In-DOTA-Tz-TCO-Pip-AR	MEAN	SD	MEAN	SD	MEAN	SD
	1 h		1 h blocking		2 h	
Blood	2.63	*0.42*	3.11	*1.55*	0.16	*0.03*
Liver	3.97	*0.86*	3.28	*1.3*	1.02	*0.38*
Intestine	0.54	*0.02*	0.4	*0.07*	0.09	*0.04*
Spleen	0.98	*0.21*	0.74	*0.42*	0.28	*0.13*
Heart	1.06	*0.26*	0.6	*0.26*	0.08	*0.01*
Lungs	1.74	*0.26*	1.44	*0.44*	0.18	*0.06*
Kidneys	9.04	*0.79*	7.18	*1.5*	3.09	*0.75*
Muscle	0.64	*0.22*	0.63	*0.29*	0.05	*0.01*
Bone	1.2	*0.43*	0.97	*0.58*	0.13	*0.06*
Stomach	0.48	*0.14*	0.34	*0.17*	0.05	*0.02*
Pancreas	3.24	*0.15*	1.67	*0.94*	0.28	*0.04*
Tumor	2.79	*0.53*	1.38	*0.53*	1.13	*0.36*
Excretion	63.2	*3.3*	60.7	*9*	91.7	*2.4*

Table S4. Biodistribution data of [^{111}In]In-DOTA-Tz in PC3-xenografts bearing mice at 15 min, 1 h and 24 hours p.i. (data expressed as mean % I.D./g and SD, n = 3).

111In-DOTA-Tz			MEAN	SD		MEAN	SD		MEAN	SD
			15 min			1 h			24 h	
	Blood		2.7	*0.4*		0.4	*0.2*		0.03	*0.0002*
	Liver		0.9	*0.1*		0.4	*0.09*		0.12	*0.02*
	Intestine		0.4	*0.1*		0.29	*0.08*		0.09	*0.04*
	Spleen		0.45	*0.05*		0.14	*0.06*		0.08	*0.01*
	Heart		0.9	*0.1*		0.17	*0.06*		0.02	*0.005*
	Lungs		1.4	*0.4*		0.38	*0.1*		0.07	*0.02*
	Kidneys		4.4	*0.5*		2.1	*0.7*		0.87	*0.14*
	Muscle		0.8	*0.4*		0.1	*0.03*		0.021	*0.005*
	Bone		0.8	*0.2*		0.2	*0.1*		0.04	*0.01*
	Stomach		0.4	*0.2*		0.17	*0.06*		0.03	*0.01*
	Pancreas		0.42	*0.02*		0.09	*0.06*		0.03	*0.01*
	Tumor		2.46	*0.6*		0.5	*0.2*		0.13	*0.04*
	Excretion		61.2	*8*		88	*4.2*		97.3	*0.9*

Table S5. Biodistribution data of the GRPR pre-targeting approach by injection of [^{111}In]In-DOTA-Tz 4h p.i. of AR-PEG4-TCO (50 µg) in PC3-xenografts bearing mice at 15 min and 1 h (with and without blocking) p.i. (data expressed as mean % I.D./g and SD, n = 3).

111In-DOTA-Tz + AR-PEG4-TCO			MEAN	SD		MEAN	SD		MEAN	SD
			15 min			1 h			1 h blocking	
	Blood		5.3	*0.75*		0.96	*0.2*		1	*0.5*
	Liver		1.65	*0.3*		0.865	*0.1*		1.2	*0.3*
	Intestine		0.84	*0.185*		0.475	*0.05*		0.4	*0.1*
	Spleen		1.2	*0.3*		0.495	*0.03*		0.4	*0.1*
	Heart		2.05	*0.55*		0.33	*0.008*		0.51	*0.03*
	Lungs		3.75	*0.95*		0.63	*0.07*		1.1	*0.2*
	Kidneys		5.75	*0.41*		3.1	*0.2*		2.5	*0.5*
	Muscle		1.65	*0.15*		0.34	*0.002*		0.24	*0.01*
	Bone		1.6	*0.5*		1.07	*0.001*		0.3	*0.03*
	Stomach		1.35	*0.55*		0.25	*0.008*		0.16	*0*
	Pancreas		1.2	*0.4*		0.4	*0.11*		0.25	*0.05*
	Tumor		3.74	*0.135*		1.19	*0.2*		0.64	*0.1*
	Excretion		32.4	*6.6*		74.65	*18.3*		84.3	*5.7*
